# Changing Technologies of RNA Sequencing and Their Applications in Clinical Oncology

**DOI:** 10.3389/fonc.2020.00447

**Published:** 2020-04-09

**Authors:** Ye Wang, Michael Mashock, Zhuang Tong, Xiaofeng Mu, Hong Chen, Xin Zhou, Hong Zhang, Gexin Zhao, Bin Liu, Xinmin Li

**Affiliations:** ^1^Clinical Laboratory, Qingdao Central Hospital, The Second Affiliated Hospital of Medical College of Qingdao University, Qingdao, China; ^2^Department of Pathology & Laboratory Medicine, UCLA Technology Center for Genomics & Bioinformatics, Los Angeles, CA, United States; ^3^Cancer Hospital of China Medical University, Liaoning Cancer Hospital and Institute, Shenyang, China; ^4^Academy of Medical Engineering and Translational Medicine, Tianjin University, Tianjin, China; ^5^Qiqihaer First Hospital, Qiqihar, China

**Keywords:** RNA sequencing, bulk RNAseq, LCM-RNAseq, single-cell RNAseq, digital spatial profiling, spatial transcriptomics, fourth-generation RNAseq, next generation sequencing

## Abstract

RNA sequencing (RNAseq) is one of the most commonly used techniques in life sciences, and has been widely used in cancer research, drug development, and cancer diagnosis and prognosis. Driven by various biological and technical questions, the techniques of RNAseq have progressed rapidly from bulk RNAseq, laser-captured micro-dissected RNAseq, and single-cell RNAseq to digital spatial RNA profiling, spatial transcriptomics, and direct *in situ* sequencing. These different technologies have their unique strengths, weaknesses, and suitable applications in the field of clinical oncology. To guide cancer researchers to select the most appropriate RNAseq technique for their biological questions, we will discuss each of these technologies, technical features, and clinical applications in cancer. We will help cancer researchers to understand the key differences of these RNAseq technologies and their optimal applications.

## Bulk RNAseq

**Figure 1:**
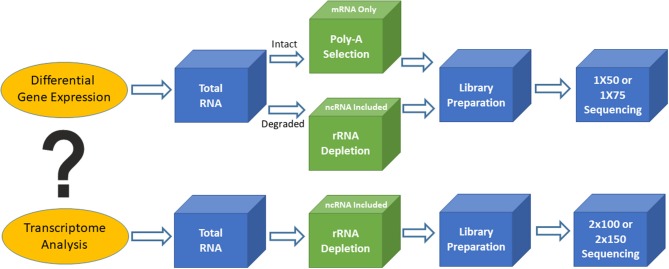
Outline of two different types of RNA sequencing. Top is for differential gene expression and bottom is for transcriptome analysis.

Since Bulk RNAseq was developed over a decade ago (1), it has become a popular genomic tool in the life science field and is shaping nearly every aspect of our understanding of genomic functions (2). Bulk RNAseq is used in >60% of all next-generation sequencing projects, including whole genome sequencing (WGS), whole exome sequencing (WES), MethySeq, chromatin immunoprecipitation sequencing (ChIP-seq), and ATAC-seq. It is the most widely used genomic technique for studying the transcriptional landscape and altered molecular pathways in human cancers. RNAseq consists of four key steps: total RNA extraction, library construction, sequencing, and data analysis. The biological question and RNA quality will dictate the type of library employed, the selection of kits, sequencing type, and sequencing depth (Figure 1).

Based on the biological questions of the researcher, there are two types of bulk RNAseq that may be employed. The first type is simple RNAseq analysis aimed at identifying differentially expressed genes or markers (signatures), in order to understand molecular mechanisms implicated in various biological processes or to guide for diagnosis and treatment. Single-read sequencing (1 × 50 or 1 × 75) is appropriate for these types of RNAseq experiments, and 20–30 million reads/sample is usually a sufficient read depth. The majority of the libraries for these purposes are prepared using the poly-A RNA selection approach. The second type of bulk RNAseq is transcriptome sequencing, which not only achieves the goals of simple RNAseq, but also extends our knowledge of alternative splicing, point mutations, novel genes and transcripts, long non-coding RNAs, and fusion transcripts. Transcriptome analysis requires paired-end sequencing (2 × 100 or 2 × 150) at 40–50 million reads/sample from each direction, and the libraries are usually prepared using the rRNA depletion approach. The ENCODE guidelines (https://www.encodeproject.org/) provide various technical details for bulk RNAseq methodology and should be used for standards to assist in designing clinical RNAseq experiments with suggestions on sequencing depth, read length, replicates, and so on.

Bulk RNAseq is a cost effective and efficient tool for both cancer research and clinical applications (3–5). Today, clinical RNAseq is mainly used in novel gene fusion discoveries, panel-based accompanying gene fusion diagnosis, whole transcriptome-based biomarker (signature) discovery, and guidance for therapeutic treatment. A good example for using RNAseq data to detect novel, and clinically relevant, gene fusions involved in cancer used large-scale transcriptome analysis (6). The study employed an in-house developed bioinformatics pipeline to detect kinase gene fusions using nearly 7,000 cancer samples from The Cancer Genome Atlas. The study had immediate clinical implications as it led to the discovery of numerous novel and recurrent kinase gene fusions, for many of which approved or exploratory drugs now exist. Several other studies have provided additional evidence to support the discovery of novel gene fusions that have benefited directly from existing kinase inhibitors or new therapeutic opportunities (7–9). For example, whole transcriptome sequencing discovered novel FGFR gene fusions that subsequently led to the development of clinical trials of the tyrosine kinase inhibitors ponatinib and BGJ398, for the treatment of cancer patients with FGFR fusions (10).

The RNA-based gene fusion detection panel was one of the first RNAseq applications successfully translated into routine clinical practice (11). The Foundation One Heme is such a clinically validated panel, an integrated DNA/RNA profiling platform using targeted next-generation sequencing. This panel includes 265 genes frequently involved in gene fusions in various cancers, including FLT3, NPM1, CEBPA, BCRABL1, KIT, IDH2, IDH1, JAK2, MPL, PML-RARA, and MLL. The gene fusions detected by RNA sequencing can be validated by targeted DNA sequencing included in the Heme panel. The test can be used by physicians to identify targeted therapy options, detect alterations for prognosis, and sub-classify sarcoma diagnoses. Another clinically used popular gene fusion panel for a companion diagnosis is the Lung NGS Fusion Profile offered by NEO Genomics. This RNA-based next-generation sequencing panel detects translocations and fusions of six genes (ALK, NTRK1, NTRK2, NTRK3, RET, and ROS1) with known and novel fusion partners. Point mutations in select exons of these six genes are also frequently detected. In non-small cell lung carcinoma (NSCLC), the gene fusions of ALK, NTRK, RET, and ROS1 are detected with the approximate frequencies of 4–6, 1, 1–2, and 1–2%, respectively. Patients harboring such gene fusions may respond to several specific kinase inhibitors.

Another key clinical application of RNAseq is the discovery of biomarkers using whole transcriptome analysis. These biomarker signatures are used for cancer diagnosis, prognosis, and prediction. The clinical utility of gene expression signatures, developed by use of microarray, QRT-PCR, and other classic methods, have been well-established and used widely in routine clinical practice, including MammaPrint, OncotypeDX, and Prosigna for breast cancer, GeneFx for lung cancer, Prolaris for prostate cancer, and ColoPrint for colon cancer. The above commonly used, clinically validated signature panels can be potentially translated into RNAseq signature panels. In fact, translatability has been demonstrated by comparing gene expression signatures in breast cancer between Affymetrix microarray and Illumina RNA-sequencing technology (12). In addition, systematic evaluation of RNAseq-based and microarray-based technology demonstrated that RNAseq is better in characterizing the transcriptome of cancer, and similar in clinical endpoint prediction, when compared with arrays. Zhang et al. (13) and Tom Lesluyes et al. (14) also used RNAseq technology with formalin-fixed paraffin-embedded (FFPE) tissue, a clinically more accessible sample type. This helped validate a prognostic signature of metastatic soft tissue sarcomas (CINSARC), developed using microarray with frozen tumor tissue, further demonstrating that CINSARC is a platform and material independent prognostic signature for metastatic sarcomas. Recently, some other RNAseq-based signatures have been developed and validated, such as the diagnostic signature for thyroid cancer (15), prognostic signatures for both Neuroblastoma (16), and Lung Adenocarcinoma (17), and predictive signatures for metastatic melanoma (18, 19).

Whole transcriptome RNAseq can also be used for guiding therapeutic treatment. Its feasibility and clinical utility in cancer were established in an early study of integrative clinical sequencing (whole exome and transcriptome analyses), which involved youths with relapsed or refractory cancer. This study identified potentially actionable findings in 46% of patients, some of those consequently changed treatment and genetic counseling (20). Later, Ronbinson et al. (21) demonstrated the broad utility of transcriptomic data in characterizing metastatic tumors and cancer treatment.

The RNAseq–based analyses have many advantages over DNA-based or other classic methods for clinical applications, including precise detail about base pairs, the ability to detect splicing variants, allele-specific expression, novel gene fusion, non-coding RNA, and novel RNAs. It is anticipated that RNAseq data will provide a more complete view of cancer-related genetic alterations and there is ample evidence to support such a view. For example, RNAseq identified an alternative breast cancer 1 (*BRCA1*) transcript in a subset of patients with breast cancer that was missed by conventional genomic analysis (22); Cabanski et al. (23) discovered a receptor tyrosine kinase (ROS1) gene fusion involved in a novel fusion partner with TMEM106B that was overlooked by standard FISH or PCR approaches; RNAseq analysis found that the germline allele-specific expression (ASE) of the transforming growth factor-beta (TGF-beta) type I receptor (TGFBR1) is associated with an increased risk of colon cancer (24); and RNAseq-based studies also show Long non-coding RNAs and miRNAs have prognostic potential in lung squamous cell carcinoma (25) and adenocarcinoma (26).

RNAseq can be applied to all tumor sample types including tumor cell lines, fresh or frozen tumor tissues, FFPE tumor tissues, and even liquid biopsy samples. The most accessible clinical tissue is FFPE tissue, which normally produces a limited amount of degraded RNA. This reality poses a challenge to produce high quality RNAseq data. Although RNAseq can reveal a more complete picture of genomic alterations in cancer, it is less commonly used compared to DNA sequencing in the clinical environment as RNA is less stable. With further technological advances, such as the development of new RNA preservative reagents, extraction methods, and RNA capture/hybridization protocols, the major hurdle of RNA stability will be overcome and the great potential of RNAseq in precision oncology will be fully realized.

It is important to keep in mind that bulk RNAseq reveals only an average gene expression profile from the studied tissue. Most tumors contain heterogeneous cell populations, including malignant cells, immune cells, fibroblasts, and vascular cells. This heterogeneity exists not only in the same tumor types from multiple patients, but also within various tumors from individual patients. The resulting average gene expression profile from tumor tissue can potentially weaken the true signals from a specific cell type that may drive tumorigenesis or resistance to treatments. For example, bulk RNAseq may have low detection sensitivity for biomarker discovery when the markers are only present in a specific cell type. This weakness can be addressed by alternative RNAseq technologies discussed in the following sections. The sensitivity issue for the companion diagnosis of gene fusion panels can be alleviated by increasing sequencing depth.

## Laser Capture Micro-Dissected RNAseq

**Figure 2:**
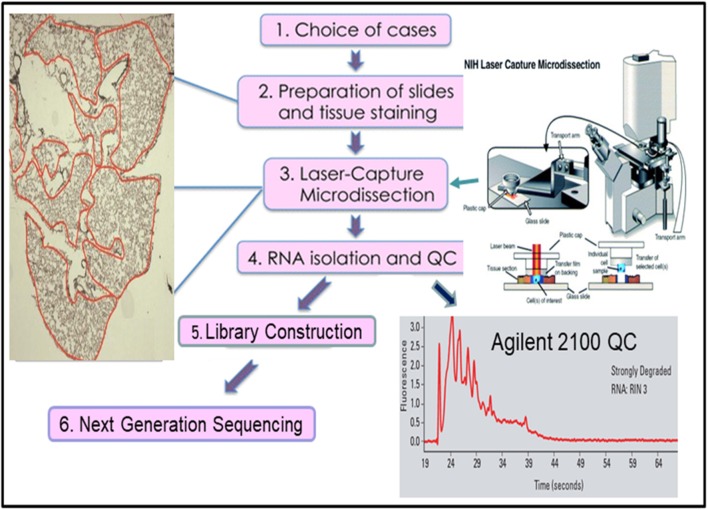
The workflow of laser capture micro-dissected RNA sequencing.

Many approaches have been developed in attempts to overcome the weaknesses of bulk RNAseq. One of the simplest approaches is laser capture micro-dissected RNAseq (LCM-RNAseq) (27). The key procedures of LCM-RNAseq consist of laser capture micro-dissection of cells of interest, followed by normal RNAseq, as illustrated in Figure 2. The majority of LCM-RNAseq employs FFPE materials, however the RNA extracted from FFPE materials are notoriously low quantity and quality and the LCM procedures further reduce the RNA integrity. As such, it is necessary to optimize LCM-RNAseq workflow by considering two critical factors: (1) optimizing the LCM procedures to minimize damage of RNA, which includes proper selection of the LCM instrument with IR laser, and (2) using a suitable RNAseq library construction kit that is optimized for the limited amount of degraded RNA (27), such as the SMARTer Stranded Total RNA-Seq Kit v2 (pico input mammalian; 250 pg−10 ng RNA input). This kit has a built-in CRIPR/CAS9-mediated rRNA depletion procedure without an independent rRNA depletion step. The kit is capable of depleting rRNA with only picograms of degraded RNA, which is not possible with library construction kits that employ a separate rRNA depletion step (normally requires >100 ng of RNA).

As the vast resource of clinical samples are FFPE tissues, further refining and improving existing LCM-RNAseq protocols could have far reaching impacts in both retrospective studies and current clinical testing of tumor samples. Recently, several new LCM-RNAseq methods were developed to address the constraints of the low input of degraded RNA derived from LCM FFPE tissues. By improving pre-amplification procedures, Singh et al. (28) claimed that sequencing data derived from as few as 10 LCM isolated single cells can reliably and sensitively measure cell-state heterogeneity in tumor tissues. A new LCM- Smart-3SEQ method was also developed that can quantify transcript abundance better with a low amount of degraded RNA from LCM cells (29). FFPEcap-seq is another method specifically designed for sequencing capped 5′ ends of RNA derived from FFPE samples. This 5′ capped RNAs-based method can also detect enhancer RNAs that arise from distal regulatory regions in addition to accurately capturing mRNA expression levels (30).

Due to technological limitations, the applications of LCM-RNAseq in cancer is relatively limited. The first application of LCM-RNAseq in cancer was performed in the normal-AIS-invasive adenocarcinoma progression model of lung cancer (31). The study established initial feasibility of LCM-RNASeq in a six patient sample population with an aim to identify biomarkers for lung cancer progression. Several studies have recently emerged, here we provide one example that highlights how LCM-RNAseq data can be used to deconvolve multiple cell type-specific gene expression profiles in cancer (32). In this study, the expression profiles of six specific tissue compartments of human glioblastoma (BGM) were analyzed using LCM-RNAseq techniques. These different compartments have interconnected complex networks and create a complex micro-environment that constantly gives signals to activate cell migration and promote cancer cell survival and proliferation (33). By isolating cell-specific gene expression signatures from different compartments, the authors found an overexpression of proangiogenic genes and pathways in pseudopalisading astrocytes cells. These overexpressed genes and pathways were known to promote cell survival and infiltrative growth, migration, and resistance to cancer-targeted therapies in GBM. Civita et al. (32) also observed a considerable up-regulation of growth factors signaling pathways in pseudopalisading cells compared to the tumor core. The data demonstrate that certain molecular events are region specific and different regions are molecularly interrelated. These findings provide potential targets for the development of new treatments and change current clinical management of BGM patients.

Although LCM-RNASeq can reveal cell population-specific gene expression profiles, it is associated with two practical issues. First, the procedure is time consuming and one can only work on a small number of cells at a time, thus data derived from 10 to 100 cells are generally less robust compared to the data obtained from >1 × 10^6^ cells of Bulk RNASeq. Secondly, the RNA yield is of low quantity and highly degraded, which requires more PCR cycles for amplification and thereby leading to poor quality RNAseq data with high PCR duplicates and possibly a biased gene expression profile. These issues need to be addressed by further technological improvement or alternative technologies. Interestingly, He et al. (11) develop a new algorithm (ADVOCATE) by using LCM-RNASeq derived data from the malignant epithelium and stroma of pancreatic ductal adenocarcinoma (PDA). These LCM-RNASeq derived algorithms can predict the compartment–specific expression profiles from bulk RNASeq profiles of PDA. This pilot work provides a framework for potentially analyzing the cellular heterogeneity of cancer and expanding the utility of the large collection of bulk gene expression data.

## Single-Cell RNAseq

There are a number of different technologies available today for single-cell RNAseq (scRNAseq) (34). The Fluidigm C1 microfluidics system represents one of the early scRNAseq technologies. This system can process only 96 single cells in a single run over 1 day. The throughput is increased to 800 single cells by using improved Fluidigm IFC chips (35). Recently, microdroplet-based single-cell sequencing systems have become dominant players. Among those, the 10x Genomics Chromium® system is one of the most popular systems for high-quality scRNAseq. In contrast to LCM-RNAseq, the Chromium technology enables rapid analysis of the gene expression profiles of up to 10,000 individual cells in one experiment.

**Figure 3:**
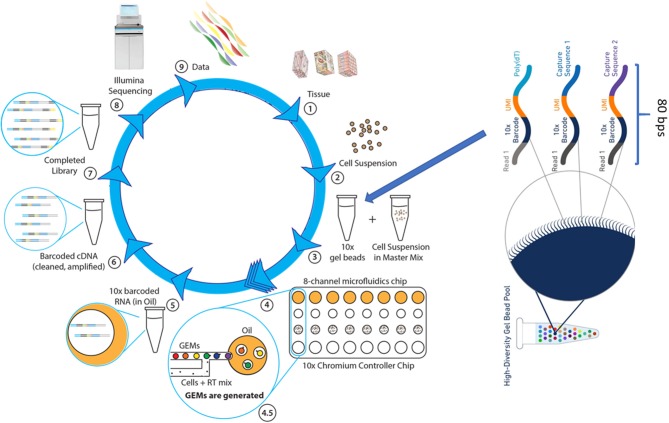
The single cell RNASeq. (1) The dissociation of tissue cells and removal of dead cells and cell debris, (2) Viable cells are resuspended in the desired buffer at a correct concentration. (3) Cell suspension is combined with RT reagents and, along with gel beads and immersion oil, introduced into Chromium Controller chip. (4) Microfluidics chip generates single cell GEMs, a gel bead bound to a cell's RNA molecules. (4.5) Gel beads and cell suspension, in RT mix, are pushed into the immersion oil. (5) GEMs are transferred into PCR tubes and undergo RT-PCR to produce cDNA. (6) The cDNA, suspended in oil, is released from GEMs, removed from oil, and amplified via PCR. (7) Libraries are completed by fragmenting the cDNA to proper insert size, followed by end repair, A-tailing, and ligation of Illumina read 2 index, all occurring in a single PCR step. (8) Sample-specific index are added and the sequence-ready libraries are sequenced by using Illumina sequencer (NextSeq 500, HiSeq3000/4000 or NovaSeq6000). (9) The 10x single cell data analysis pipeline employs Cell Ranger to align reads and perform cluster and gene expression analysis, followed by Cell Loupe Browser to visualize and analyze the Cell Ranger data output.

The Chromium scRNAseq workflow, similar to other microdroplet scRNAseq systems, is illustrated in Figure 3. The first critical step for scRNAseq is to isolate viable, individual cells from targeted tissues or cultured cells. Then, the Chromium microfluidics system is used to inexpensively generate hundreds of thousands of microdroplets, called GEMs, which are aqueous microdroplets surrounded by oil. Each GEM has a volume of ~2 nl that includes all necessary reagents for reverse transcription (RT) and also contains a bead conjugated with a specific 80-base pair (bp) oligo sequence. This oligo sequence has several components, including adaptor sequences for next-generation sequencing (NGS) (Read 1), a cell-specific 10x barcode for identifying which cell the RNA comes from, the random molecular tags for identifying and quantifying unique mRNA transcripts [i.e., unique molecular identifiers (UMI)], and polyoligo-dT primers for mRNA binding. Following cell lysis, the oligo-dT on the beads hybridizes to the poly-A tail of the released mRNA, and then RT reactions are carried out within the microdrops. At this step, the bead-specific oligo sequence is incorporated into the cDNA, which is used to align sequence reads back to a specific cell. GEMs are then broken, cDNAs are pooled together, amplified, and purified, followed by NGS library preparation using 10x Genomics protocols. The libraries can be sequenced by using Illumina sequencers at the following settings with Novaseq 100 cycle sequencing kit: 28 bps for read 1 (sequencing the cell-specific 10x barcode), 8 bps for i7 (sequencing the sample barcode), and 91 bps for read 2 (sequencing single-cell RNA). The 10x Genomics Chromium system and its associated library construction kits are commercially available. This system offers significant advantages over other microfluidics systems, such as Dropseq and Fluidigm C1, primarily in data quality and throughput. Therefore, it has become an important analytical tool for researchers in many disciplines, particularly in cancer research.

One key feature of tumors is their heterogeneity. The analysis of bulk RNAseq is complicated by significant infiltration of stroma and other type of cells in the tumor. Given the quantitative nature of gene expression data, it can be difficult to deconvolve the functionally relevant signals from average signals derived from bulk RNAseq. The scRNAseq technology offers a complementary and powerful tool to dissect intratumoral transcriptomic heterogeneity (36), important for therapeutic response. A good example of such is an early study on drug resistance in a model of drug tolerance with a metastatic breast cancer cell line. By analyzing untreated, stressed, and drug-tolerant cell groups, authors demonstrated that drug-tolerant cells contain specific RNA variants in genes involved in microtubule organization, stabilization, cell adhesion and cell surface signaling (37). This drug-tolerant-specific RNA variants were absent in untreated or stressed cells. The generation of specific RNA variants increases heterogeneity and ensure the survival of a minority population that efficiently converse stress-tolerant cells back to normal cells. Single cell analysis can also provide insightful clue for tumor treatment. Due to the intratumoral heterogeneity, a given targeted therapy often eliminates a specific subpopulation of tumor cells while leaving others unharmed. To overcome this challenge, therapeutic strategies that can target multiple tumor subpopulations are critical. By analyzing numerous drug target pathways in various cell populations in metastatic renal cell carcinoma, Kim et al. used scRNAseq technology to successfully develop an optimized combinatorial therapeutic strategy that showed significantly improved response *in vitro* and *in vivo* compared to monotherapies (38).

Another major application of single cell sequencing is to characterize known cell types, subtypes, and previously unknown cell types within and surrounding tumors, and to identify the gene signature for given cell types (39–41). These studies have facilitated dissection of complex pathways in heterogeneous tumor tissues and have provided guidance for cancer treatment. Here, we highlight how single cell sequencing technology was used to identify new cell types and biomarkers in T cell infiltration. The status of T cell infiltration and their characteristics are associated with different prognostic outcomes (42) and it is important to the development of immunotherapies and the prediction of their clinical responses in cancers. In 2017, Zheng et al. (40) performed a comprehensive analysis of infiltrating lymphocytes in liver cancer and reveals distinctive functional composition of T cells in hepatocellular carcinoma (HCC). The study identified 11 large subsets as well as unique subpopulations, such as CD8^+^FOXP3^+^ regulatory-like cells and clonal TCRs, at single-cell level. They also identified *LAYN*, an HCC-associated Treg marker gene, which is associated with tumor-infiltrating exhausted CD8^+^ T cells and poor prognosis. The authors have made this comprehensive single T cell database publicly available for the wide research community (http://hcc.cancer-pku.cn).

Today, single-cell transcriptomic analysis has revolutionized our understanding of cancer biology, including tumor heterogeneity and their therapeutic implications. However, the major limitation of the technology is the level of detail that can be resolved from the captured mRNA data. Although the 10X genomics chromium system can capture up to 10,000 cells in a single experiment, it can only recover a few thousand unique transcripts from a single cell. By deeper sequencing, this problem can be alleviated to a certain degree, but is still far less than ideal for full transcriptome analysis. In conclusion, bulk RNAseq, LCM-RNAseq, and single-cell RNAseq all suffer from a common weakness—lost critical spatial information due to the micro-dissection or cell dissociation at the early stage of these protocols, which impacts the understanding of cell functionality and pathological changes (43). These limitations can be addressed by recently developed spatial profiling technologies as discussed below.

## Digital Spatial Profiling

Each organ of a complex organism consists of diverse cell types that often interact in highly structured manners under distinct microenvironments. Such highly structured spatial heterogeneity enables the organism to function correctly and efficiently. To fully understand gene functions in a given cell type, one must study gene expression in the context of the location of the cells in the tissue (44). However, none of the technologies discussed above can provide this critical spatial information. Traditionally, immunohistochemistry and *in situ* hybridization have been used to reveal spatial gene expression in tissue sections, but the throughput of these procedures is limited to the analysis of only one or a few genes at a time.

**Figure 4:**
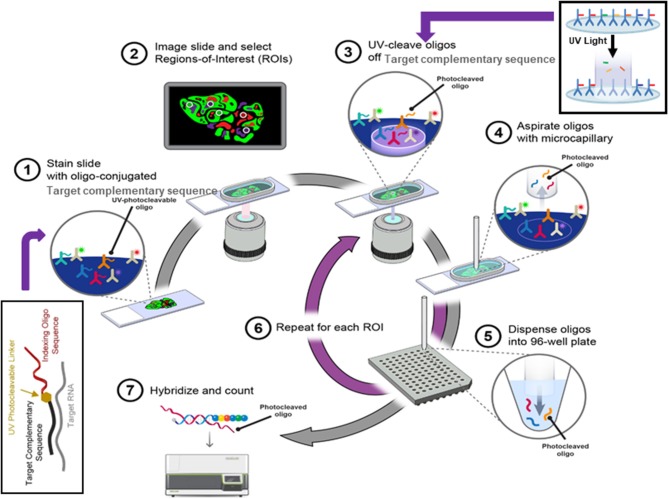
The digital spatial profiling. (1) Apply high-plex oligo-labeled probes to FFPE slide. (2) Use visible wavelength low-plex imaging to establish tissue “geography.” Select regions-of-interest (ROIs) for high-plex profiling. (3) UV-release oligo tags at selected ROIs. (4, 5) Collect and dispense released tags in microtiter plate. (6) Repeat the procedures for each ROI. (7) Index, hybridize, and count the tags per ROI and analyze the data with nSolver™ Advanced Analysis Software.

Recently developed digital spatial profiling (DSP) technology has made it possible to resolve spatial gene expression with significantly improved throughput. DSP is based on the nCounter® barcoding technology from NanoString Technologies to enable spatially resolved, digital characterization of mRNA expression in a highly multiplexed assay (up to 1,000 RNA targets). The key technology of the assay relies upon RNA hybridization probes conjugated to photo cleavable oligonucleotide tags. After binding of probes to their targeted mRNA on the slide-mounted FFPE tissue sections, the slide is imaged. Then, the oligonucleotide tags are released from the regions of interest on the tissue via UV exposure. Released tags are quantified in an nCounter® assay. The counts of a specific tag, representing a specific mRNA, are mapped back to tissue location (defined region of interest), yielding a spatially-resolved digital profile of mRNA abundance (Figure 4).

The GSP technology from NanoString Technologies, called GeoMx DSP, is commercially available and has already shown valuable applications in elucidating tumor microenvironments, immuno-oncology biomarker discovery, and optimizing immunotherapy by using targeted small gene panels. Ihle et al. (45) characterized tumor microenvironment of lytic and blastic bone metastases in prostate cancer patients using DSP technology, and found a distinct set of immune cell populations and signaling pathways specifically present in lytic or blastic types of prostate cancer. The immune cells in blastic lesions were enriched for pSTAT3 and JAK-STAT pathway related genes while pAKT activity and PI3K-AKT pathway related genes were more active in lytic-type lesions. The direct implication of this finding is that the targeted therapies for pAKT or pSTAT3 can potentially be considered. In addition, the immune checkpoints, such as PD-L1, were identified in blastic prostate cancer, which can now be considered as a new therapeutic target for blastic prostate patients with bone metastases.

Two landmark studies demonstrated that DSP is also a powerful tool for biomarker discoveries and optimizing therapeutic strategy (46, 47). In these studies, authors demonstrated that the combined ipilimumab and nivolumab therapies had high response rates with more lymphoid infiltration, whereas treatment with nivolumab monotherapy had modest responses with a more clonal and diverse T cell infiltration in responders, respectively, and that low RNA expression of the IFN-γ signature was associated with relapse after combinational therapies (ipilimumab + nivolumab), while none of the patients with a high or intermediate IFN-γ signature has relapsed in high-risk melanoma patients (47). Both studies identified promising biomarkers for further validation and offered possible solutions for optimizing immunotherapy strategy.

The DSP technology is becoming popular in the digital gene expression space. We expect that more applications of this technology in clinical oncology will emerge in the next few years. However, the relatively small number of mRNA targets that can be investigated simultaneously, the requirement of pre-knowledge of the gene, and inability to reveal sequencing information have limited its applications on a broader scale. Given that spatial gene expression is critical for understanding cell identity and function in tissue content, there is compelling reason to expect innovation to continue in the field.

## Spatial Transcriptomics

A new spatial transcriptome technology has been in active development for several years. This technology overcomes the limitations of DSP technology by allowing scientists to study the whole transcriptome spatially (43). It can theoretically provide information similar to bulk transcriptome analysis along with spatial content.

**Figure 5:**
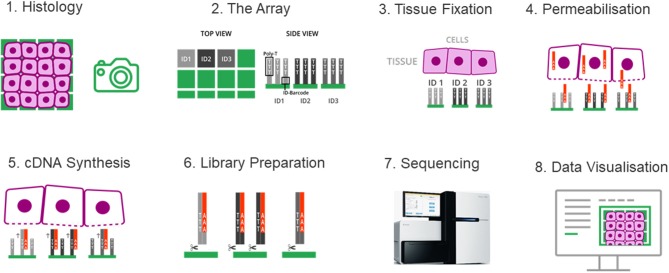
The spatial transcriptomes. (1) A freshly frozen tissue section is prepared and attached onto the chip. (2) The chip contains an array of distinguishable capture probes. The Poly-T tails of these capture probes can bind the Poly-A tails of RNA molecules. (3) The tissue section is fixed and imaged, which makes it possible to overlay the cell tissue image and the gene expression data in a later step. (4) The tissue is permeabilized and RNA molecules can exit the cells through small holes created in the cell membrane, and bind to the adjacent capture probes on the chip. (5) cDNA synthesis is performed on the chip. (6) The cDNA-RNA-hybrids are cleaved off the chip, followed by library construction. (7) The libraries are sequenced. (8) Data are visualized to determine where genes are expressed and in what quantity.

Spatial transcriptomics adopted a strategy that integrates the features of microarray and the barcoding system of 10x Genomics. Briefly, a fresh-frozen tissue section is imaged and placed on a patterned, barcoded oligo-dT microarray slide. The capture probes on the slide include a T7 promoter for *in vitro* transcription (IVT), a partial Illumina handle for the sequencing, a spatial barcode for RNA localization, a UMI for removing amplification duplicates, and oligo-dT sequences for capturing mRNA. The tissue is then fixed and permeabilized to release RNA, which binds to adjacent oligo-dT sequences of capture probes. During the cDNA synthesis, the spatial barcodes indicating the location of each spot on the array are incorporated into the cDNA. One strand of double stranded cDNA, which contains the information of where the cDNA came from, is cleaved off from the array. The libraries are completed off the chip and then sequenced. The spatial barcode allows each read to be mapped to the correct spatial coordinates (Figure 5). Since spatial transcriptomics technology displays spatially-resolved whole transcriptome data on the original tissue section, scientists can choose all or any number of genes of interest to visualize and analyze.

The spatial transcriptomic analysis is generally applicable to fresh-frozen mammalian tissues and fresh plant tissues (48), and is potentially applicable to FFPE materials (44). The application of this powerful technology in the cancer arena is still limited and only tested in early technology access. One critical application is to investigate intratumor heterogeneity, which has posed a challenge to understanding tumor progression and treatment. Dr. Joakim Lundeberg's group has used this technology to explore the landscape of tumor heterogeneity in prostate cancer (49) and melanoma (50). By profiling 6,750 and 2,200 tissue regions in prostate and melanoma, respectively, they showed extraordinary gene expression heterogeneity between biopsies (distinct gene expression signature) and different regions within the biopsy (coexistence of several expression profiles) (50). The gene expression heterogeneity extends well-beyond cell type or tissue type. For example, the lymphoid area adjacent to the tumor region had a specific expression pattern (50), non-tumor tissue in close proximity to the tumor region displayed a gradient expression pattern and unique cancer expression profiles progress beyond pathologically defined tumor boundaries (49). All these findings suggest that the location-dependent gene expression is a reflection of cell-cell interactions in tumor microenvironment and certainly impact immune cells' function and therapy response. This is a key area that deserves further investigation in order to fully understand tumor metastasis.

Spatial transcriptomics technology has also been used in cancer diagnosis. Yoosuf et al. (51) used publicly available breast cancer spatial transcriptomics datasets, in combination with machine learning technique, to distinguish ductal carcinoma *in situ* (DCIS) from invasive ductal carcinoma (IDC). By identifying spatial transcriptomics signatures from known DCIS and IDC regions and training the machine learning method, they achieved a prediction accuracy of 95% for DCIS and 91% for IDC. This pilot study demonstrates the power of spatial transcriptomics in breast cancer diagnosis and subtype characterization.

The spatial transcriptomics technology does not require specialized equipment or pre-knowledge of gene sequences, and has a throughput higher than that of digital spatial profiling methods. The limitation of this technology is that the currently available product is not able to offer single-cell resolution as it is limited by the microarray spot size and spacing. However, in October, 2019, Stahl's group claimed that they have improved the spatial resolution of this technology by 1,400x so that it can now study spatial gene expression at the single cell level, opening the opportunity to detect tumor cells in the critical early stages (52). In today's clinical management of oncological patients, we need to quickly identify resistant clones during standard targeted therapies and discover robust, sensitive biomarkers to predict response to immunotherapy. Given the current resolution and sensitivity of spatial transcriptomics technology, this is an ideal technology to resolve these unmet needs.

## Fourth-Generation RNAseq

The ultimate goal of RNAseq is a simple, robust, spatially-resolved transcriptomic analysis at a single-cell resolution. The recent developments of fourth-generation sequencing technologies, such as *in situ* sequencing (ISS) and fluorescent ISS (FISSEQ), have potential toward this final destiny (36, 52). The detailed technologies, promises, and consequences were reviewed by Ke et al. (53). The ISS method applied padlock probes combined with rolling circle amplification (RCA) to generate *in situ* amplified, targeted sequencing libraries that are subsequently sequenced via sequencing-by-ligation NGS chemistry (53). Through sequencing of a molecular barcode, consisting of four bases in the non-target hybridization part of the padlock probes, the ISS method can simultaneously sequence up to 256 unique transcripts. As this method uses target-specific padlock probes to create rolling circle amplification products, it is used only for sequencing known genes, such as gene panels. In contrast, the fluorescent *in situ* sequencing (FISSEQ) method uses random hexamers with a sequencing primer tag to initiate *in situ* RT. Different from cDNA in ISS, the resultant cDNAs are circularized using CircLigase. During RT, dUTP is introduced and the cDNAs are cross-linked to tissue with the reagent BS (PEG)9 to prevent diffusion of the cDNAs. After RCA, the products are sequenced by using the same sequencing by ligation techniques. By applying FISSEQ with a 30-base read length, Lee et al. obtained 156,762 reads covering 8,102 annotated genes in human primary fibroblasts (36).

Compared to ISS methods, FISSEQ generates random libraries and, in principle, allows an unbiased analysis of all cellular transcripts at a single-cell resolution. Practically, the number of transcripts detected in each cell is low (54), since the majority of sequenced molecules are rRNAs. In this regard, ISS technology uses targeted gene panels and thus the sensitivity of ISS is around two orders of magnitude higher than that of FISSEQ for any given gene (55).

Although ISS and FISSEQ technologies each have their own strengths in detection, these technologies are still in their very early developmental stages and many technical aspects need to be addressed before they can be applied in cancer research and clinical applications. The main bottlenecks are tissue preparation, optimized methods for improving efficiency, computational tools, and imaging scale. However, fourth-generation RNAseq provides a direct *in situ* sequencing approach. If technical obstacles can be addressed in the coming years, fourth-generation RNAseq can potentially become a straightforward method for high-throughput spatial transcriptomic analysis.

## Summary

Among six RNAseq technologies described above, each has its own strengths, weaknesses and suitable applications, as summarized in Table 1. We anticipate that bulk RNAseq will remain the primary choice for clinical oncology in the near future, the application of single cell sequencing will further expand when it becomes more cost-effective, and technologies with spatial content will be the final destiny in precision oncology.

**Table 1:** Key strengths, weaknesses, and current suitable applications of six RNASeq technologies in clinical oncology.

	**Strengths**	**Weaknesses**	**Suitable applications**
Bulk RNASeq	High throughput, cost effective, mature technology	Average gene expression profile, lack of spatial content	Whole transcriptome-based biomarker discovery, targeted RNAseq panel for gene fusion
LCM-RNAseq	Cell type specific gene expression profile	Time consuming, low quality data, lack of spatial content	Tumor heterogeneity by dissecting cell type specific population
Single cell RNASeq	>10,000 single cell gene expression profile	High cost, a limited number of unique transcripts, lack of spatial content	Tumor heterogeneity, cell type characterization, and discovery
Digital spatial profiling	Spatial information, applicable to FFPE materials	Limited to small number of genes (gene panel only), lack of sequencing information	Tumor microenvironments, immuno-oncology biomarker discovery and optimizing immunotherapy
Spatial transcriptomics	Whole transcriptome analysis with spatial and sequencing information	Long procedures, early stage of technology	Tumor heterogeneity, tumor microenvironments, optimizing immunotherapy
Fourth generation RNAseq	*In situ* sequencing with future potential	In-matured technology	Not demonstrated yet

## Author Contributions

YW and XL conceived and designed the project. MM and XZ wrote the part of bulk RNAseq. ZT and HC wrote the part of laser capture micro-dissected RNAseq. XM and HZ wrote the part of single cell RNAseq. GZ and BL wrote the part of digital spatial profiling. YW wrote the whole manuscript.

### Conflict of Interest

The authors declare that the research was conducted in the absence of any commercial or financial relationships that could be construed as a potential conflict of interest.
